# Reading and Calculation Neural Systems and Their Weighted Adaptive Use for Programming Skills

**DOI:** 10.1155/2021/5596145

**Published:** 2021-08-04

**Authors:** Joao Castelhano, Isabel C. Duarte, Joao Duraes, Henrique Madeira, Miguel Castelo-Branco

**Affiliations:** ^1^CIBIT/ICNAS, University of Coimbra, Coimbra, Portugal; ^2^CNC.IBILI, Faculty of Medicine, University of Coimbra, Coimbra, Portugal; ^3^CISUC-DEIS, Polytechnic Institute of Coimbra, Coimbra, Portugal; ^4^CISUC-DEI, University of Coimbra, Coimbra, Portugal

## Abstract

Software programming is a modern activity that poses strong challenges to the human brain. The neural mechanisms that support this novel cognitive faculty are still unknown. On the other hand, reading and calculation abilities represent slightly less recent human activities, in which neural correlates are relatively well understood. We hypothesize that calculus and reading brain networks provide joint underpinnings with distinctly weighted contributions which concern programming tasks, in particular concerning error identification. Based on a meta-analysis of the core regions involved in both reading and math and recent experimental evidence on the neural basis of programming tasks, we provide a theoretical account that integrates the role of these networks in program understanding. In this connectivity-based framework, error-monitoring processing regions in the frontal cortex influence the insula, which is a pivotal hub within the salience network, leading into efficient causal modulation of parietal networks involved in reading and mathematical operations. The core role of the anterior insula and anterior midcingulate cortex is illuminated by their relation to performance in error processing and novelty. The larger similarity that we observed between the networks underlying calculus and programming skills does not exclude a more limited but clear overlap with the reading network, albeit with differences in hemispheric lateralization when compared with prose reading. Future work should further elucidate whether other features of computer program understanding also use distinct weights of phylogenetically “older systems” for this recent human activity, based on the adjusting influence of fronto-insular networks. By unraveling the neural correlates of program understanding and bug detection, this work provides a framework to understand error monitoring in this novel complex faculty.

## 1. Introduction

Software programming is a complex and phylogenetically very recent human activity (100 years old), even more than reading/literacy (which started around 5000 BC) or complex mathematics (3000 BC) [1]. Importantly, in a century where computers dominate, there is an increasing interest in understanding the neural correlates of program comprehension [2].

The transversal nature of software use (i.e., software is needed for almost all modern human activities) makes software development one of the largest industry sectors, if not the largest. However, in spite of decades of research and advances in software engineering and software reliability, software defects (i.e., bugs) remain as the most enduring problem of software quality [3–5]. The average number of bugs per 1000 lines of delivered code (KLOC) [6] remains astonishingly high, which reinforces the importance of understanding error-monitoring processes in the brain during execution of this novel complex task. This might have important implications for understanding how the brain can control programming performance [7].

In neuroscientific terms, there is the debate [2] if programming requires the expert integration of mathematical and language skills, including logical thinking and symbol manipulation. Programming may require a large set of skills beyond mathematical calculations using numbers and might require integration with the language/reading skills at an abstract level. In this line, the reading and cognitive analysis of algorithms likely requires a large set of regions with distinctive contributing weights for optimal performance.

The functional anatomy of reading and language [8, 9] has been studied for many years [10–13]. There is evidence for a task-dependent connection between language, reading, and arithmetic/calculation skills from both behavioral [14–17] and imaging studies [18, 19]. The interaction of these networks during programming tasks remains to be investigated. However, concerning the neural correlates of computer program reading and understanding, functional neuroimaging studies are still scarce due to the inherent challenges in performing such studies [20, 21]. The relation between error-monitoring and program understanding processes and how information is integrated between reading-related regions—including the visual word form area [22] and middle temporal, inferior, and middle frontal gyrus regions [23]—and calculation processing networks—involving bilateral parietal regions, including the precuneus [24]—remain elusive. To clarify how does the brain effectively utilize these circuits, and their relative weights, for effective programming remains a very interesting question. In this work, we try to understand the particular patterns of recruitment of reading and calculation circuits for programming, as a function of their coordinated enrolment by high-level neural systems.

The interplay between brain networks involved in syntactic processes, arithmetic operations as recursion, or language processing are known [25, 26]. Language, in particular reading, and calculation may require processing across common neural networks [27] possibly because recursive processing and/or symbolic operations are important across these cognitive domains. Whole-brain imaging suggested nevertheless a near-complete spatial separation of areas activated by calculation and reading [28, 29] and, e.g., a separation between code and prose writing [30, 31]. The use of those networks for computer code understanding might represent an instance of the “reutilization/recycling” hypothesis [1].

To perform programming tasks, it is likely that the brain needs to “reutilize” brain networks in an adaptive manner for this type of complex activity, possibly by reorganizing this form of complex integrative processing in a top-down manner [1]. Dehaene et al. have pioneered this “recycling” account whereby cortical regions may be partly recycled for new human-specific uses. In other words, a brain region that evolved for a given processing demand might be reutilized in novel ways and distinct weights when new demands emerge during human history for a given new function [22].

In spite of the evidence for a network involved in programming skills [7, 30, 32, 33], this does not necessarily imply a novel form of brain specialization but might emerge from a new form of top-down controlled brain connectivity with distinct weights of reading and calculation systems.

In this study, we aimed at understanding which are the common and separable brain networks supporting both calculation and reading processes in adults [31] and what are their relative weights in programming-related tasks. We used evidence from the current data-driven meta-analysis and additional review of emerging neuroimaging of programming literature to suggest that program understanding recruits error-monitoring fronto-insular circuits which integrate (weighted) resources from processing modules related to visual, language, reading, calculation, and memory processing [34–36] and put forward a new theoretical framework that should be tested in the future.

## 2. Materials and Methods

We investigated into which extent activity across regions involved both in reading and math operations supports the more recently evolved cognitive process of program understanding. To address this question, we took advantage of a meta-analysis quantitative approach [37–39], a strategy that allows for the identification and localization of brain regions exhibiting commonalities (the main focus for hypothesis generation) and differences across tasks [40, 41]. The integration of neuroimaging data is important because it allows overcoming limitations due to small sample sizes, which limit generalization [42]. To demonstrate the possible interdependence of activation of regions involved in reading, calculation, and program understanding, we further compared the results of the meta-analysis with the results from the emerging literature of fMRI studies of programming.

### 2.1. Study Design and Review Protocol

We performed 2 meta-analyses, which were carried out using the activation likelihood estimation (ALE) analysis, one including data published in neuroimaging studies of reading and the second with studies of calculation. We performed a contrast and conjunction analysis of reading and calculation studies (suppl. Table A1 and A2) following the PRISMA guidelines in meta-analysis [43]. Moreover, we compared the results with the emerging and recently published literature on program understanding.

### 2.2. Search Strategy and Data Sources

We performed the literature search using the BrainMap (Sleuth 2.4) database. The Sleuth search criteria for reading were as follows: “*Diagnosis* is Normal and *Stimulus* is visual and *Imaging modality* is fMRI and *Paradigm class* is Reading and *Activation* is activations only.” The search criteria for calculation were as follows “*Diagnosis* is Normals and *Stimulus* is Visual and *Imaging* modality is fMRI and *paradigm class* is Counting/calculation and *Activation* is activations only.” The programming-related fMRI studies were manually found from each of the reference list of the different papers published in the field. Supplementary Figure A1 (PRISMA) summarizes the number of articles and duplicates that were found. The final study included 68 reading and 73 calculation studies (Supplementary Table A1 and A2, respectively) and 7 programming studies. We then used these foci of brain activations for the ALE analysis. These data provide an effect size Cohen's *d* = 0.8845.

To identify functional brain imaging studies, our inclusion criteria were as follows: (1) the studies imaged the whole brain (studies reporting only ROI analysis were excluded); (2) the results presented coordinate-based data in a standard space; (3) experimental paradigms included visual stimuli, a reading task (words, pseudowords, or sentences, the instruction being to read), calculating (and/or arithmetic operations) tasks, or programming-related tasks (source-code understanding; bug detection; and code writing); (4) the imaging method was fMRI, and only activations were considered; (5) subjects were healthy controls; and (6) sample size *N* ≥ 8 [39].

The supplementary material includes the PRISMA figure, the tables reporting the papers included and the individual meta-analysis results, and the figure representing the superimposed results from the different meta-analysis results.

### 2.3. Data Extraction

We exported data as a text file containing all the coordinates of the results from the original publications for the three conditions. All coordinates were converted between Talairach/MNI standard spaces (using the Brett transform as implemented in the mni2tal or tal2mni function of MATLAB (v2013a, MathWorks, USA).

### 2.4. Data Analysis

We applied the activation likelihood estimation (ALE) method to reading, calculation, and programming fMRI studies, using data published in healthy control subjects (see supplementary figure A1). This method entails a coordinate-based meta-analysis (CBMA) of whole-brain studies [42, 44–47]. A 3D Gaussian function is used at each coordinate with a certain FWHM, which depends on the sampling size, and a nonparametric test is performed against a null hypothesis derived from permutation analysis [39]. The ALE algorithm uses a random-effects model, which is more conservative than the fixed-effects model. It incorporates modeling of both within and between study variance to minimize the possibility that the results might be influenced by a possible variability of the included studies [42].

The ALE meta-analysis was carried out as described previously by [44]. A permutation (1000 permutations) statistical test of randomly distributed foci was computed to assess the statistical significance of the results including a family-wise error rate (FWE) threshold set to *P* < 0.05 and a minimum cluster size of 200 mm^3^ [39]. We used GingerALE, the Java version of ALE developed at the Research Imaging Center and available at http://brainmap.org/ale for data processing.

To determine the differences between the ALE maps for reading and calculation, the two meta-analysis studies were pooled and contrasted using the GingerALE software. We followed the work described in [48]. In this contrast analysis, new-threshold (*P* < 0.05) ALE images are created using a voxel-wise minimum statistic [49] by contrasting the individual ALE images (already FWE corrected for multiple comparisons). In order to take into account the differences between studies included in the meta-analysis and to obtain a voxel-wise *P* value image, we performed a 1000 permutation analysis using a *P* value of 0.05 and a minimum volume of 200 mm^3^ [39]. The resulting ALE contrast images were converted to *Z* scores in order to simplify interpretation and show their significance. The same procedure was applied to compare reading and calculation with programming studies.

For visualization, the results were overlaid into an image of the International Consortium for Brain Mapping single-subject MRI anatomical template in the MNI space [50]. GingerALE tools were used to convert results between Talairach and MNI spaces.

### 2.5. Review of fMRI Studies of Programming Skills

To further evaluate the hypothesis that program understanding shares, the same resources as reading and/or calculation, one takes into account the results from the available fMRI studies using a program understanding task [7, 24, 30, 32, 33, 51–53]. These are, to our knowledge, the only studies available in the literature about the neuronal correlates of program understanding. The one from Castelhano et al. uniquely reported functional and effective connectivity, but the amplitude findings of the available articles provide relevant insights on the relative weight of each network in programming tasks [2, 30, 32, 33]. The work from Siegmund et al. used detection of syntax errors as contrast condition to investigate the cognitive process of programming/source-code comprehension. The others focused on specific processing neural mechanisms requiring program understanding, because they required the identification of bugs in computer code, which requires deeper program understanding. On the other hand, the 2020 work from Krueger et al. was focused on code writing. They found that code writing involves the right hemisphere brain regions involved in spatial ability and planning and present evidence suggesting that code and prose writing are quite dissimilar at the neural level. Ikutani et al. [33] showed a fine-tuned representation of source code in the brain while Ivanova et al.'s work [32] report code comprehension activations in particular differences in BOLD responses to code problems with responses to content-matched sentence problems. All the available fMRI studies of programming (a still new field with relatively few studies (*N* = 7)) are included in the meta-analysis (including contrast and conjunction comparisons with reading and calculation). This last particular analysis is exploratory given the limited sample size [39].

## 3. Results

**Figure 1:**
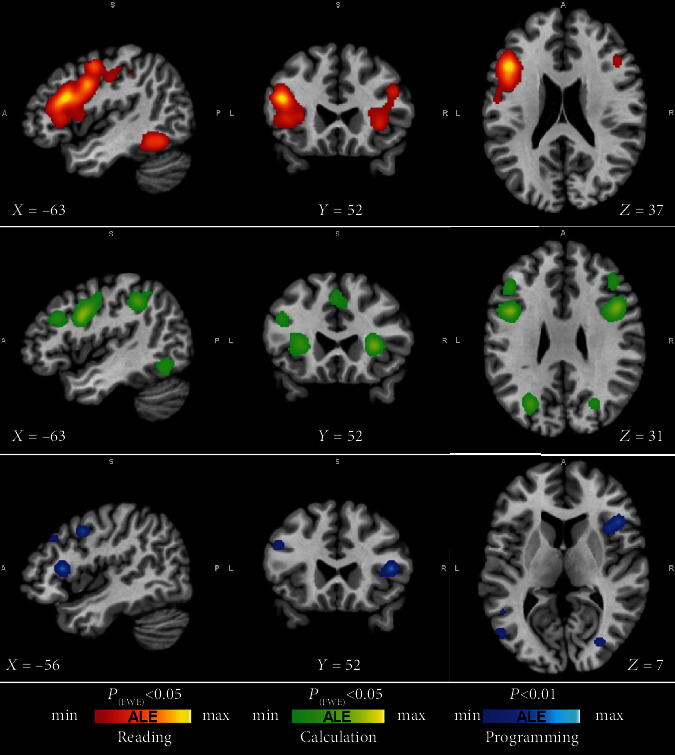
Brain activation maps of reading, calculation, and programming skills. The ALE results corrected with FWE are shown for the three conditions in separate panels (suppl. figure A2 report the superimposed maps). Frontal decision-related areas and other calculation (parietal precuneus), reading (middle temporal including visual word form area and inferior frontal gyrus), and insula regions are activated during programming tasks.

We performed the ALE meta-analysis and distinct contrast studies using the activation data to compare reading, calculation, and programming neural correlates. The individual meta-analysis results of brain activation associated with each of these conditions are shown in Figure 1 and detailed in supplementary material (Table A3). We found reliable activations across reading studies spanning a ventro-temporal and frontal network. Regarding calculation, the analysis revealed mainly a parieto-frontal network. Accordingly, our review of the literature investigating programming shows that previous works included 166 subjects (mean age range: 20-28 years) and revealed a network of areas, some of which overlap with the areas identified for the other conditions, in particular the frontal region BA6, the anterior insula, and the parietal regions.

Most importantly, we asked which regions *jointly* activated regarding calculation/math tasks and reading and found that these include particularly a set of frontal (BA6, BA9, and BA10) and the superior parietal regions involved in executive function and the anterior insula (Tables 1 and 2). In this line, we found regions in the frontal gyrus, parietal lobule, insula, and occipital gyrus, possibly representing a network involving fronto-insular-parietal connections. This meta-analysis therefore helped us define in a data-driven manner (corroborating our own previous model-driven study of code comprehension) a core set of regions-of-interest involved in programming, which also validates the choice derived from our previous study [7]. Moreover, a conjunction analysis of programming and calculation shows common activation mainly at the middle frontal and precentral gyrus (BA6, BA19, and BA46) and the insula (BA13) both at left and right hemispheres. On the other hand, we found middle frontal gyrus and middle temporal gyrus activations (BA6, BA9, BA13, BA20, BA21, BA37, and BA46) for programming and reading conjunction analysis.

The contrast analysis between these conditions (Table 2) shows that calculation activated more the inferior parietal lobule (BA40) than reading or programming for both hemispheres. Regions most activated for reading vs. calculation or reading vs. programming represent a temporal-frontal network mainly at the left hemisphere. The comparison between programming and calculation studies reveals higher activations for programming at middle temporal regions (mainly for the left, BA20, BA21, and BA22) and middle frontal regions (BA6, BA8, BA9, BA45, and BA46) both on the left and right hemispheres. Regarding the programming vs. reading comparison, programming tasks activated more frontal, insular, and temporal regions while reading has increased the activity at the superior temporal, inferior, and medial frontal gyrus and cingulate gyrus. These might represent a parieto-temporo-frontal network comprising BA2, BA6, BA8, BA9, BA13, and BA21.

Additionally, a closer look into the programming studies identified a set of regions (Figure 1; Table 3; Figure A2) involved either in reading, calculus/math, or both: Brodmann areas 6, 21, 39, 40, 44, and 47 [51, 52, 54]. The medial frontal cortex, including the cingulate cortex and, most importantly, the anterior insula were also activated [7, 24, 30, 32, 33]. In the study by Castelhano et al., (blue regions represented in Figure 1) we investigated the neural underpinnings of programming by using fMRI while subjects performed a bug-detection task, which requires deep program understanding. This study revealed a brain network that includes the above-mentioned regions of the saliency network (cingulate cortex and insula) related to error monitoring, dorsolateral middle frontal and other regions involved in working memory and executive function, and posterior regions, namely superior parietal. Others have reported that prose writing entails significant differences when compared to code writing: prose writing activates left hemisphere regions associated with language, while code writing preferentially recruits the right hemisphere, including regions associated with attention control, working memory, planning, and spatial cognition [30], which might be further specialized for the domain of programming [33]. The relation with attention control and working memory may not be specific to programming, at least in part, but these functions are particularly engaged in this complex task.

## 4. Discussion

We first hypothesized that program understanding is jointly dependent, but with different weights, on processing of calculus/math operations and reading skills, which motivated the meta-analysis, including conjunction approaches, to identify critical hubs and to test if they converge with the ones identified in the emerging literature on neuroimaging of program comprehension. Using this strategy, we identified a functional architecture underlying this cognitive function.

**Figure 2:**
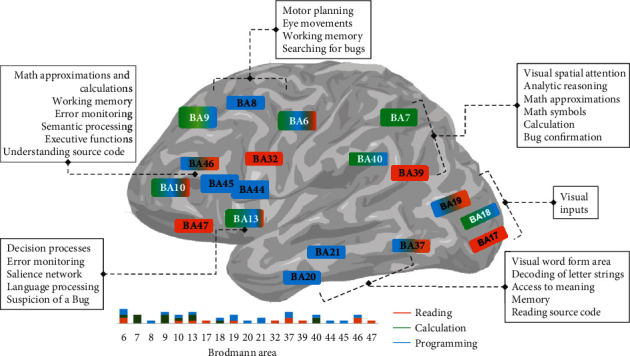
A schematic representation of the reading and calculation systems and their weighed use for programming skills in the brain. Previous works suggest the circuitry that is recruited for tasks requiring these skills. Regions shown in orange, green, and blue increase their activation for the reading, calculation, and programming tasks, respectively (numbers indicate the Brodmann areas). A series of brain regions link and share resources for the distinct tasks (bar plot summary represent the normalized number of studies reporting those regions). Despite the fact that ventro-temporal areas related to reading are visible, the parietal regions related to calculation and visuo-spatial attention are strongly recruited under programming demands. The summary boxes indicate the known functions per region, and in bold, the function we suggest that those regions might be involved for particular programming skills.

The involvement of frontal decision-related areas, error-monitoring regions such as the insula and cingulate cortex and other calculation (parietal precuneus) regions are in line with our prediction that an integrated system recruiting areas associated to other tasks such as reading, working memory, and calculus operates during program understanding [2]. In particular, attention and planning processes involving parietal regions related to the processing of calculus are activated (BA7, BA40), in line with the hypothesis that earlier regions involved in mathematical and reading operations are recruited for programming, albeit with distinct weights (Figure 2).

From our review, multiple cognitive processes seem to be required with distinct weights: cognitive analysis of algorithms and code language as well as mental calculation and working memory for operations such as multiplication and sorting. These weights may be dependent on whether the programming is dominantly graphical or not [32]. The regions subserving such weight-dependent integration mechanisms are required for calculus/math, reading, or both, as identified in our meta-analysis. These regions include decision-related areas in frontal cortex and other math (parietal precuneus) and the anterior insula. Reading-related regions (middle temporal including visual word form area and inferior frontal gyrus) may also be activated during program understanding tasks [32, 33]. In fact, our results show a close overlap of the reading regions involved in processing language (extended frontal and middle temporal activations) with those needed for computer programming skills. Since their behavioral types appear to be conceptually related, this relation is expected. However, the observed lateralization patterns, which are not merely a reflection of language lateralization, suggest that additional computational processes kick in during programming tasks. Moreover, attentional, mental imagery, and manipulation of symbols strongly recruit the right hemisphere [1, 16, 30, 55–57]. Although attention control and working memory may not be specific to programming, they are particularly engaged in this complex task. Furthermore, we found common patterns of activation for calculation and programming skills in the middle frontal and precentral gyrus and the insula. These results plausibly confirm our hypothesis of recruitment of shared resources between those complex skills. Although reading and calculation share the same type of hemispheric dominance and may have partly shared the same primitive computational mechanisms [58], and in particular recursion, it is known that linguistic/semantic and mathematics skills do not necessarily share the same brain architecture of causally directed influences [28, 31]. The language processing (in particular reading regions) is important to computer programming. Moreover, calculation competences are also needed to understand programming. In fact, these are required combined skills to learn programming [59, 60]. A connection between language and arithmetic has been suggested in both behavioral [14, 16, 17] and imaging studies [18, 19, 61], in line with recent models of complex mental processing [62]. This hypothesis of shared resources and distinct connectivity in the brain might also work as a basis network for programming. In this line, we suggest that emerging connectivity patterns might play a role in programming skills but our view is limited by the nature of the works available to this review.

Recent studies show that the general semantic system (e.g., language) responses during code comprehension are relatively more weak and inconsistent [32] but might play a role in learning to process computer code [63–65]. This is expected due to the nature of specific programming demands that recruit those networks only into a certain extent depending on task requirements (and in general in a more limited manner for the reading network). For example, types of functions such as bug-specific error-monitoring processes or mathematical recursivity (related to programming loops) may require particular processing requirements. We identified with the contrast and conjunction analyses common signatures between those skills. This is in line with the notion that basic mathematical and reading skills are needed before any learning of programming abilities can be successfully initiated [59, 60]. Each of the identified neuroimaging studies regarding programming revealed clusters that were also reliably activated in other studies assessing phonological processing and calculation tasks [31, 41]. This matches the models of complex mental processing that suggest the use of shared resources in the brain to deal with this kind of complex skills [62] in particular for the representation of amounts symbolically or quantitatively [16] and associated with executive load and selective attention [66] or symbol recognition and processing of multiple words and digits.

We found also a fronto-insular-parietal network [35, 36] suggesting a pivotal role for central executive and salience networks. It is important to highlight the recent work that showed these brain regions have enough information to decode functional categories of source code [33] that, in line with our previous work on the role of the insula, show correlated activity with individual behavioral performance in code inspection and bug detection. Previous studies focused only in the identification of bugs in computer code [7, 24, 54]. Now the role of programming writing is also beginning to be under scrutiny and might be useful to further understand the neural underpinnings of overall programming skills [30]. Requesting participants to search for bugs in the code can only partly help disentangle the brain regions activated during understanding program code at a deep level. Such bug-detection mechanisms are probably related to activation in the anterior insula that is known to be associated with decision and error monitoring [67], and as part of the salience network [68, 69] or the error and novelty processing in the anterior cingulate. The relative roles of the insula and anterior cingulate within the saliency network remain a topic of hot debate, which concerns the relation with task difficulty and error monitoring. We posit that the insula is more directly related to decision as a function of task difficulty, as suggested by previous work [7, 70, 71]. It is possible that insular contributions may be considered generically evaluating the quality of evidence that might be relevant for a decision on code quality and accuracy [7]. This is consistent with the notion that the insula does belong to the salience network which is associated with cognitive control mechanisms that support arithmetic processing [72], source-code debugging, and decision-making. The insula or part of the cingulate cortex is also involved in other processes such as cognitive saliency and emotion. Thus, during these bug-detection tasks, the insula might be activated not only due to error processing but also because of the engagement of high cognitive processes related to saliency detection or even emotional/reward responses (e.g., frustration for not finding the bugs in the code).

**Figure 3:**
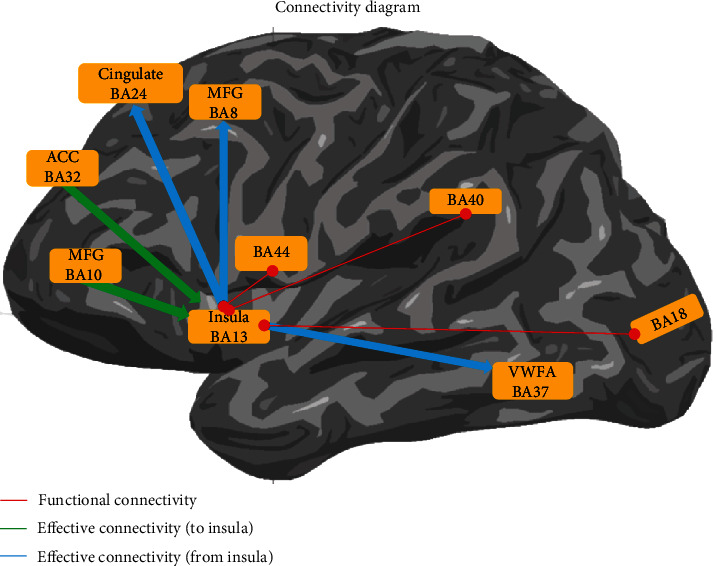
Network analysis summary in a form of directional diagram. A previous work revealed the insula receives directed input from the anterior cingulate BA32 and middle frontal gyrus BA10 and gives directed input to the frontal regions in cingulate gyrus BA24 and middle frontal gyrus BA8. Moreover, the functional integration also includes a path to other sensory/visual areas (BA18) or math processing regions (BA40/BA44) [7].

Based on these results, we suggest a novel network architecture related to programming tasks and in particular bug detection in the brain. This overlapping network, which includes fronto-insular and parietal regions (depicted in the conceptual framework in Figure 3), is also supported by our previous evidence showing that connectivity between frontal regions, the insula and parietal math processing regions (possibly related to the first insight of the algorithm in the source code), cooccurs with directed interactions (effective connectivity) to reading regions. Given that programming is a far more complex skill set than reading or calculation, we suggest that this complex network emerges as a function of task demands, whereby distinct weights of those cognitive modules are pivotal in that set (Figure 3). Interestingly, a new form of top-down controlled brain connectivity with distinct weights of reading and calculation systems might be important.

These modules are organized as follows: frontal regions related to math operations, working memory, error monitoring, semantic processing, and executive functions tend to be more activated in program understanding tasks [7, 51, 53]. Other medial frontal (cingulate cortex) and insular areas related to the salience network are required for deeper levels of program understanding [30, 32, 33] that are needed to error monitoring. Indeed, the insula might be activated here as part of a more general neural architecture of error processing and novelty in the anterior midcingulate cortex [73, 74].

Our analysis revealed that parietal regions related to calculation and visuo-spatial attention are activated under programming skill requirements, in addition to concurrent recruitment of ventro-temporal areas related to reading. Connectivity studies further corroborating the proposed conceptual framework will be needed in the future.

## 5. Conclusion

Our data-driven theoretical proposal suggests that computer programming skills rely on differential weighted recruitment of reading and calculation networks, fueled by a pivotal contribution of the anterior insula hub within the saliency network. This might have important implications for shedding light on how the brain can improve programming performance by improving such “reutilization” of earlier processing modules/networks. This opens the path to a neuroscience-informed approach that may allow establishing predictive relationships between brain activity and computer programming skills. The discussion about the reutilization based on connectivity changes might be pivotal to understand the brain architecture that is recruited during programming and should benefit from studies with subjects learning programming as a new skill.

## Figures and Tables

**Table 1:** Overlap in brain activation across studies, as assessed using a conjunction analysis of reading, calculation, and programming. The major activation is shown with their corresponding Brodmann area (BA), the ALE value of the peak-activated voxel, and MNI coordinates. Statistical criteria were *P* < 0.05 with 1000 permutations and a minimum volume of 200 mm^3^.

Task	Cluster	Peak coordinates			ALE	Hem.	Lobe	Region	BA
		*X*	*Y*	*Z*					
Reading and calculation	1	-45.71	7.69	29.27	0.158	L	Frontal	Inferior frontal gyrus	9
*P* (FWE) < 0.05 (1000 permutations)		-47.97	23.91	19.01	0.123	L	Frontal	Middle frontal gyrus	46
Cluster threshold > 200		-47.7	0.41	45.51	0.094	L	Frontal	Precentral gyrus	6
		-31.02	24.75	0.9	0.094	L	Sublobar	Insula	13
	2	-28.27	-56.74	47.85	0.102	L	Parietal	Superior parietal lobule	7
		-45.45	-37.98	44.25	0.084	L	Parietal	Inferior parietal lobule	40
		-28.34	-72.92	31.37	0.079	L	Occipital	Precuneus	31
		-26.18	-70.45	35.6	0.078	L	Parietal	Precuneus	19
		5.73	13.94	50.46	0.122	R	Frontal	Superior frontal gyrus	6
		-4.97	13.7	48.37	0.121	L	Frontal	Superior frontal gyrus	6
		-2.69	-2.35	60.84	0.066	L	Frontal	Medial frontal gyrus	6
	4	31.57	-54.27	46.96	0.122	R	Parietal	Superior parietal lobule	7
	5	-43.69	-61.63	-14.01	0.11	L	Temporal	Fusiform gyrus	37
		-39.29	-80.12	-5.82	0.069	L	Occipital	Inferior occipital gyrus	19
	6	48.32	10.36	27.96	0.108	R	Frontal	Inferior frontal gyrus	9
		46.06	33.27	21.61	0.069	R	Frontal	Middle frontal gyrus	9
	7	35.23	22.86	-1.93	0.116	R	Sublobar	Insula	13
	8	-26.41	-92.56	-2.69	0.075	L	Occipital	Inferior occipital gyrus	18
	9	48.63	-34.79	49.59	0.073	R	Parietal	Inferior parietal lobule	40
	10	-26.24	-3	54.48	0.074	L	Frontal	Middle frontal gyrus	6
	11	33.41	-90.26	-5.8	0.064	R	Occipital	Inferior occipital gyrus	18
	12	37.48	-63.77	-21.45	0.065	R	Cerebellum	Posterior lobe	

Programming and calculation	1	-30.3	-73.51	26.59	0.008	L	Occipital	Superior occipital gyrus	19
*P* < 0.01 (1000 permutations)	2	-42.42	-2.78	36.85	0.008	L	Frontal	Precentral gyrus	6
Cluster threshold > 200	3	-50.51	27.49	29.75	0.011	L	Frontal	Middle frontal gyrus	46
	4	38.38	16.27	5.21	0.008	R	Sublobar	Insula	13
	5	47.78	2.84	42.27	0.007	R	Frontal	Precentral gyrus	6
	6	28.28	1.03	65.35	0.005	R	Frontal	Middle frontal gyrus	6

Programming and reading	1	-42.42	-2.78	36.85	0.008	L	Frontal	Precentral gyrus	6
*P* < 0.01 (1000 permutations)	2	38.38	16.27	5.21	0.008	R	Sublobar	Insula	13
Cluster threshold > 200		44.44	22.14	12.05	0.006	R	Frontal	Inferior frontal gyrus	13
	3	-49.49	26.46	29.69	0.01	L	Frontal	Middle frontal gyrus	46
	4	-50.51	-53.54	-3.09	0.007	L	Temporal	Middle temporal gyrus	37
	5	46.46	4.22	41.58	0.006	R	Frontal	Middle frontal gyrus	9
	6	-10.1	-5.25	67.19	0.004	L	Frontal	Superior frontal gyrus	6
	7	-12.12	-3.09	65.13	0.003	L	Frontal	Medial frontal gyrus	6
	8	-56.57	-42.83	-12.01	0.003	L	Temporal	Middle temporal gyrus	20
	9	-58.59	-40.87	-9.51	0.003	L	Temporal	Middle temporal gyrus	21
	10	-6.06	-3.3	69.47	0.002	L	Frontal	Superior frontal gyrus	6

**Table 2:** Major ALE foci for the contrast study between the three conditions: programming, reading, and calculation.

Task	Cluster	Peak coordinates			*Z* value	Hem.	Lobe	Region	BA
		*X*	*Y*	*Z*					
Reading > calculation	1	-48.15	25.23	11.32	3.72	L	Frontal	Inferior frontal gyrus	47
*P* (FDR) < 0.05 (1000 permutations)	2	-40.77	-50.41	-19.22	3.72	L	Cerebellum	Posterior lobe	^∗^
Cluster threshold > 200	3	-46.5	3.56	50.37	3.72	L	Frontal	Precentral gyrus	6

Calculation > reading	1	39.74	-43.47	44.18	3.71	R	Parietal	Precuneus	7
*P* (FDR) < 0.05 (1000 permutations)		23.1	-57.86	56.28	3.54	R	Parietal	Precuneus	7
Cluster threshold > 200	2	-26.36	0.39	54.3	3.71	L	Frontal	Middle frontal gyrus	6
	3	-33.62	-45.18	50.63	3.71	L	Parietal	Inferior parietal lobule	40

Programming > calculation	1	44.75	20.09	7.372	2.88	R	Frontal	Inferior frontal gyrus	45
*P* < 0.04 (1000 permutations)	2	-52.53	-49.42	-2.857	2.37	L	Temporal	Middle temporal gyrus	22
Cluster threshold > 200	3	-57.58	-15.91	-14.03	1.71	L	Temporal	Middle temporal gyrus	21
	4	-56.57	-44.84	-13.32	2.46	L	Temporal	Middle temporal gyrus	20
	5	-13.54	-3.911	71.51	1.76	L	Frontal	Superior frontal gyrus	6
	6	-52.12	-75.26	-2.086	1.76	L	Occipital	Inferior temporal gyrus	37
	7	6.364	49.49	46.8	1.76	R	Frontal	Medial frontal gyrus	8
	8	21.82	0.923	65.34	1.76	R	Frontal	Subgyral	6
	9	-51.52	25.97	29.12	3.09	L	Frontal	Middle frontal gyrus	46
	10	51.52	25.22	33.98	1.85	R	Frontal	Middle frontal gyrus	9

Calculation > programming	1	31.72	-62.94	47.39	2.37	R	Parietal	Superior parietal lobule	7
*P* < 0.012 (1000 permutations)		33.64	-58.01	43.62	2.26	R	Parietal	Inferior parietal lobule	40
Cluster threshold > 200	2	-32.32	-57.76	42.66	2.58	L	Parietal	Inferior parietal lobule	40
		-22.93	-61.15	46.61	2.51	L	Parietal	Precuneus	7

Programming > Reading	1	23.54	1.232	65.36	1.85	R	Frontal	Middle frontal gyrus	6
*P* < 0.042 (1000 permutations)	2	-51.52	-73.82	-1.884	1.85	L	Occipital	Inferior temporal gyrus	37
Cluster threshold > 200	3	44.44	20.28	7.6	2.75	R	Frontal	Inferior frontal gyrus	13
		40.4	14.31	2.932	1.96	R	Sublobar	Insula	^∗^
	4	29.39	-81.5	5.602	1.73	R	Occipital	Middle occipital gyrus	19
	5	-62.63	-42.83	-12.01	1.98	L	Temporal	Middle temporal gyrus	21
	6	-13.03	-3.644	72.39	1.85	L	Frontal	Superior frontal gyrus	6
	7	6.162	49.25	47.54	1.85	R	Frontal	Medial frontal gyrus	8
	8	-48.79	28.45	31.21	2.88	L	Frontal	Middle frontal gyrus	9
	9	-54.75	-30.88	50.28	1.85	L	Parietal	Postcentral gyrus	2
	10	47.27	2.561	46.06	1.73	R	Frontal	Middle frontal gyrus	6
	11	-59.6	-13.01	-14.1	1.73	L	Temporal	Middle temporal gyrus	21

Reading > programming	1	-50	6.887	13.64	2.75	L	Frontal	Precentral gyrus	44
*P* < 0.05 (1000 permutations)		-34.34	20.39	5.429	2.51	L	Sublobar	Insula	13
Cluster threshold > 200		-43.33	14.02	9.011	2.37	L	Frontal	Precentral gyrus	44
		-46.46	25.64	3.53	2.23	L	Frontal	Inferior frontal gyrus	47
		-52.53	8.134	2.606	2.14	L	Temporal	Superior temporal gyrus	22
		-40.4	11.12	26.71	2.07	L	Frontal	Middle frontal gyrus	9
	2	-2.02	1.109	48.38	1.64	L	Limbic	Cingulate gyrus	24
		-6.364	4.821	50.64	1.64	L	Frontal	Medial frontal gyrus	32

**Table 3:** Regions activated during program understanding.

Programming	Threshold	*X*	*Y*	*Z*	*Z* or *T* value	Size	Hem.	Lobe	Region	BA
Ivanova et al. 2020	*P* < 0.001								Parietal regions	
									Precentral	
									Middle and inferior frontal gyrus	
									Medial frontal	
									Insula	
									Middle orbito frontal	
									Posterior temporal	

Ikutani et al. 2020	*P* (FWE) < 0.05	46	22	8	6.81	369	R		IFG (*P. triangularis*)	
		-12	0	66	5.35	298	L			
									Posterior-medial frontal	
		6	52	42	5.17	587	R		Superior medial gyrus	
		-56	-28	50	5.16	649	L		Inferior parietal lobule	
		24	4	60	4.84	428	R		Superior frontal gyrus	
		-52	30	24	4.79	346	L		IFG (*P. triangularis*)	
		-52	-72	2	4.5	347	L		Inferior occipital gyrus	
		-50	-54	0	4.35	347	L		Inferior temporal gyrus	

Krueger et al. 2020	2.1 < *t* < 6.2						L		Left post	5
									Central gyrus and superior parietal lobule	
							L		Primary motor cortex	4
							L and R		Premotor/supplementary motor cortex	6
							R		Including the superior and middle frontal gyri	09-10
							R		Inferior and middle temporal gyri	18-19
							R		Inferior parietal lobule	39-40
							R		Anterior insula	13
							L		Anterior insula	13

Castelhano et al. 2019	*P* (FDR) < 0.05	27	-66	38	10	5442	R	Parietal	Precuneus	7
		-27	-84	-1	10	4921	L	Occipital	Middle occipital gyrus	18
		48	18	35	8	2694	R	Frontal	Middle frontal gyrus	9
		-48	9	41	11	2265	L	Frontal	Middle frontal gyrus	8
		33	-84	-16	9	1514	R	Posterior	Declive	
		54	-45	-13	9	849	R	Temporal	Inferior temporal gyrus	20
		42	-45	44	7	413	R	Parietal	Inferior parietal lobule	40
Siegmund et al. 2017	*P* (FDR) < 0.01									21, 40, 44

Siegmund et al. 2014	*P* (FDR) < 0.01									6, 21, 40, 44, 47
Duraes et al. 2016	*P* (FDR) < 0.05	38	16	3			R		Insula	13

Blank spaces in the table: data not reported.

## Data Availability

Due to privacy/ethical restrictions, the data available is available on request from the corresponding author.

## Supplementary Materials

Supplementary Materials Figure S1: PRISMA 2009 flow diagram for the meta-analysis. (A) Reading papers. Among the excluded papers that did not meet the inclusion criteria, the main reasons were as follows: studies that reported only ROI analysis or did not report results coordinates in a standard space (*N* = 23; screening step); techniques other than fMRI (*N* = 17); studies of special subject populations (*N* = 3) or not adults (*N* = 4); reviews (*N* = 1), tested other brain functions, and/or did not use visual stimuli (*N* = 6); and studies with less than 8 subjects (*N* = 6). (B) Calculation papers. Among the excluded papers that not meet the inclusion criteria, the main reasons were as follows: studies that reported only ROI analysis or did not report results coordinates in a standard space (*N* = 39; screening step); techniques other than fMRI (*N* = 1); not adults (*N* = 2); reviews (*N* = 2), tested other brain functions (*N* = 19), did not use visual stimuli (*N* = 3), and the task was passive viewing (*N* = 5); studies with less than 8 subjects (*N* = 2) (Eickhoff et al. 2016). Figure S2: brain activation maps of reading, calculation, and programming skills. Frontal decision-related areas and other calculation (parietal precuneus) and reading (middle temporal including visual word form area and inferior frontal gyrus) are activated during programming tasks. Maps are represented in a standard MNI image and FWE corrected for multiple comparisons. Table S1: reading studies included in the meta-analysis. Table S2: calculation/arithmetic studies included in the meta-analysis. Table S3: major activation likelihood estimation results for the reading, calculation, and programming tasks separate analysis.
