# Supplementary material for: Reading and Calculation Neural Systems and Their Weighted Adaptive Use for Programming Skills
Source: Neural Plast. 2021 Aug 4;2021:5596145. doi: 10.1155/2021/5596145 (PMC8360733; doi:10.1155/2021/5596145)
Supplement: Supplementary Materials — Figure S1: PRISMA 2009 flow diagram for the meta-analysis. (A) Reading papers. Among the excluded papers that did not meet the inclusion criteria, the main reasons were as follows: studies that reported only ROI analysis or did not report results coordinates in a standard space (N = 23; screening step); techniques other than fMRI (N = 17); studies of special subject populations (N = 3) or not adults (N = 4); reviews (N = 1), tested other brain functions, and/or did not use visual stimuli (N = 6); and studies with less than 8 subjects (N = 6). (B) Calculation papers. Among the excluded papers that not meet the inclusion criteria, the main reasons were as follows: studies that reported only ROI analysis or did not report results coordinates in a standard space (N = 39; screening step); techniques other than fMRI (N = 1); not adults (N = 2); reviews (N = 2), tested other brain functions (N = 19), did not use visual stimuli (N = 3), and the task was passive viewing (N = 5); studies with less than 8 subjects (N = 2) (Eickhoff et al. 2016). Figure S2: brain activation maps of reading, calculation, and programming skills. Frontal decision-related areas and other calculation (parietal precuneus) and reading (middle temporal including visual word form area and inferior frontal gyrus) are activated during programming tasks. Maps are represented in a standard MNI image and FWE corrected for multiple comparisons. Table S1: reading studies included in the meta-analysis. Table S2: calculation/arithmetic studies included in the meta-analysis. Table S3: major activation likelihood estimation results for the reading, calculation, and programming tasks separate analysis. [file 5596145.f1.docx]

***Supplementary Material***

**
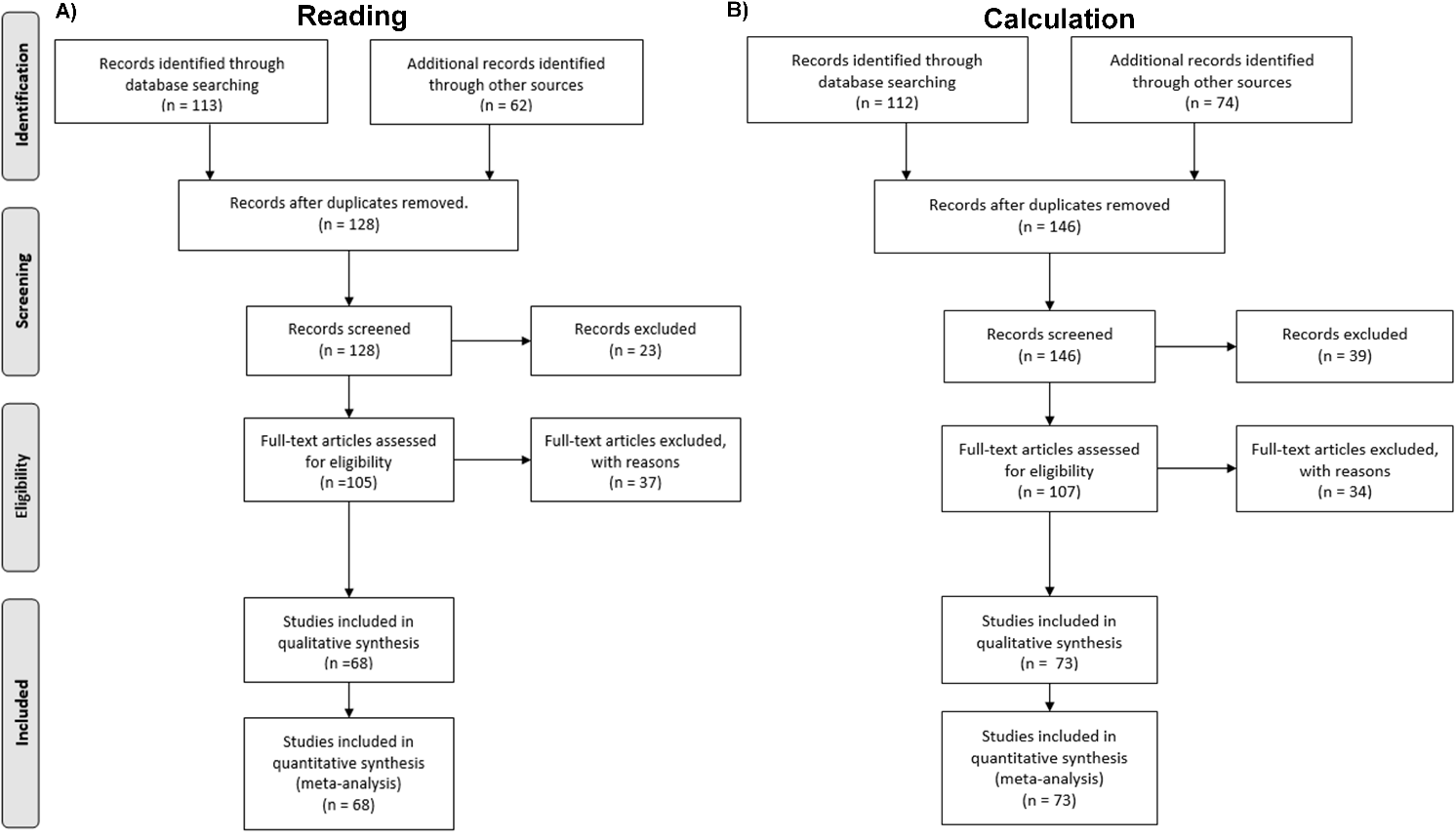
**

**Figure A1. PRISMA 2009 flow diagram for the meta-analysis. A) Reading papers.** Among the excluded papers that not meet the inclusion criteria, the main reasons were: studies that reported only ROI analysis or did not report results coordinates in a standard space (N=23; screening step); techniques other than fMRI (N=17); studies of special subject populations (N=3) or not adults (N=4); reviews (N=1), tested other brain functions and/or did not use visual stimuli (N=6); and studies with less than 8 subjects were also excluded (N=6). B) **Calculation papers**. Among the excluded papers that not meet the inclusion criteria, the main reasons were: studies that reported only ROI analysis or did not report results coordinates in a standard space (N=39; screening step); techniques other than fMRI (N=1); not adults (N=2); reviews (N=2), tested other brain functions (N=19), did not use visual stimuli (N=3), the task was passive viewing (N=5); studies with less than 8 subjects were also excluded (N=2) (Eickhoff et al. 2016).


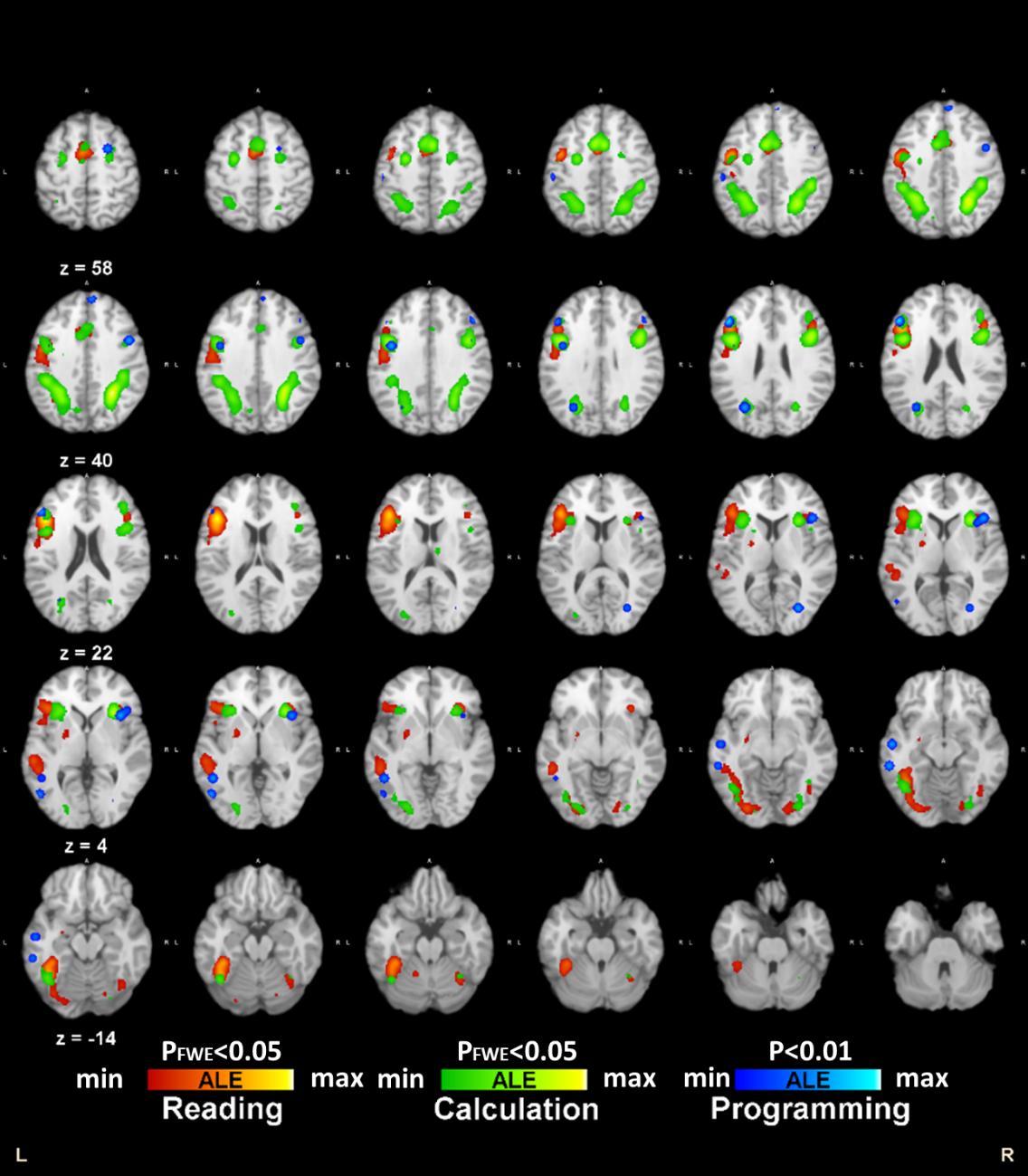


**Figure A2. Brain activation maps of reading, calculation and programming skills**. Frontal decision related areas and other calculation (parietal precuneus) and reading (middle temporal including visual word form area and inferior frontal gyrus) are activated during programming tasks. Maps are represented in a standard MNI image and FWE corrected for multiple comparisons.

**Suppl. Tables**

**Table A1. Reading studies included in the meta-analysis.**

| **Reference** | **N** | **Gender** | | **Age (years)** | | **Task** | **Foci** | **MRI vendor and Field Strength** | **Software**  **Used** | **Statistical threshold** | **TR (ms)** | **Resolution (mm)** |
| --- | --- | --- | --- | --- | --- | --- | --- | --- | --- | --- | --- | --- |
|  |  | M | F | mean | range |  |  |  |  |  |  |  |
| (Cohen et al. 2002) | 16 | 3 | 13 | 25 | 20-30 | Reading words | 14 | 1.5T Signa GE | SPM99b | voxelwise threshold p<0.01 | 3000 | 3.75 |
| (Haist et al. 2001) | 15 | 6 | 9 | 26 |  | Reading words | 7 | 1.5T Signa GE |  | Pbonferroni <0.05 | 3000 | 3.75 |
| (Aparicio et al. 2007) | 12 | 6 | 6 | 34 | 20-49 | Reading words and pseudowords | 8 | 2T S200 Bruker | SPM99 | P<0.001 corrected at the cluster level | 5000 | 2 |
| (Y. Liu et al. 2007) | 23 |  |  |  |  | Passive Reading | 28 | 3T Siemens Allegro | AFNI | level of P < 0.005. | 2000 | 3.3 |
| (Brambati et al. 2006) | 11 | 5 | 6 | 28 | 14-55 | Reading | 82 | 1.5T Horizon GE | SPM2 | voxelwise threshold p<0.005 | 3000 | 4 |
| (Uchiyama et al. 2006) | 20 | 10 | 10 | 22 | 19-29 | Reading | 36 | 3T Siemens Allegro | SPM2 | Pcorr<0.05 | 2500 | 3 |
| (Brem et al. 2009) | 18 | 9 | 9 | 25 | 20-31 | Reading words and strings | 9 | 1.5T Intera Philips | SPM5 | P(FDR)<0.01 | 3000 |  |
| (Dietz et al. 2005) | 16 | 7 | 9 | 31 | 21-40 | Reading | 7 | 1.5T Magnetom Siemens | MEDx | Punc<0.001 | 12000 | 3.6 |
| (Rapp et al. 2004) | 15 | 6 | 9 |  | 19-51 | Reading Sentences | 23 | 1.5T Sonata Siemens | SPM99 | Punc<0.001 | 2000 | 3 |
| (Suh et al. 2007) | 16 | 14 | 2 | 23 | 19-29 | Reading Sentences | 5 | 3T ISOL Tech | SPM2 | Punc<0.00001 | 3000 |  |
| (Yokoyama et al. 2006) | 36 | 30 | 6 | 21 | 18-29 | Reading Sentences | 8 | 1.5T Shymphony Siemens | SPM99 | Punc<0.001 | 4000 | 2 |
| (Ye and Zhou 2009) | 19 | 7 | 12 | 21 | 19-23 | Reading words / semantic discrimination | 4 | 3T Trio Siemens | SPM5 | Punc<0.001 | 2000 | 3 |
| (Stanislas Dehaene et al. 2001) | 37 |  |  | 23 | 19-34 | Reading (covert) | 15 | 3T Bruker | SPM99 | voxelwise threshold p<0.001 | 2400 |  |
| (Ruff et al. 2001) | 12 | 6 | 6 | 23 |  | Reading (covert) | 23 | 1.5T Horizon GE | SPM99 | Pcorr<0.05 | 3000 | 3.75 |
| (Kang et al. 1999) | 14 | 5 | 9 | 21 | 18-26 | Reading (covert) | 14 | 1.5T Signa GE | RSVP | Punc<0.05 | 1050 | 10 |
| (Vingerhoets et al. 2003) | 12 | 12 | 0 | 28 | 19-49 | Reading (covert) | 26 | 1.5T Shymphony Siemens | SPM99 | Punc<0.001 | 3000 | 3 |
| (Savige and Fontana 1976) | 9 | 2 | 7 | 60 | 42-68 | Reading (covert) | 20 | 1.5T Signa GE | SPM99 | voxelwise threshold p<0.005 | 2500 | 3.75 |
| (Tremblay and Gracco 2006) | 12 | 3 | 9 | 26 |  | Reading words and word generation | 18 | 1.5T Sonata Siemens | AFNI | minimum by bonferroni | 2880 | 4 |
| (Jobard et al. 2007) | 10 | 10 | 0 | 22 | 18-26 | Reading | 61 | 1.5T Horizon GE | SPM99 | Punc<0.001 | 6000 | 3.75 |
| (Ahrens et al. 2007) | 8 | 8 | 0 | 21 | 20-22 | Reading (covert) sentences | 48 | 1.5T Vision Siemens | MEDx/Matlab6.5 | voxelwise threshold p<0.001 | 3000 | 3 |
| (Epstein et al. 2006) | 12 | 5 | 7 | 32 | 23-53 | Reading (covert) | 2 | 3T Signa GE | SPM99 | voxelwise threshold p<0.001 | 1200 | 5 |
| (Meschyan and Hernandez 2006) | 12 | 5 | 7 | 22 | 20-25 | Reading | 18 | 3T GE | SPM99 | voxelwise threshold p<0.001 | 3000 | 4 |
| (Golestani et al. 2006) | 12 | 7 | 5 |  | 20-28 | Reading (covert) | 33 | 3T Bruker | SPM99 | voxelwise threshold p<0.001 | 2000 | 3.75 |
| (Carreiras et al. 2007) | 36 | 13 | 23 |  | 22-46 | Reading | 129 | 1.5T siemens | SPM2 | Pcorr<0.05 | 3150 | 3 |
| (Rektorova et al. 2007) | 8 | 8 | 0 | 62 |  | Reading sentences | 35 | 1.5T Shymphony Siemens | SPM99 | voxelwise threshold p<0.001 | 3800 | 1.7 |
| (Tourville, Reilly, and Guenther 2008) | 11 | 5 | 6 | 28 | 23-36 | Reading aloud | 60 | 3T Trio Siemens | SPM2 | P(FDR)<0.05 | 2000 | 3.125 |
| (Wilson et al. 2009) | 9 | 2 | 7 | 66 |  | Reading (overt) | 48 | 3T Signa GE | SPM5 | voxelwise threshold p<0.001 | 1650 | 3.4 |
| (Kerr et al. 2004) | 14 | 6 | 8 | 21 | 19-24 | Reading (overt) | 22 | 3T Allegra Siemens | |  | 5000 | 3 |
| (Hauk et al. 2008) | 21 | 11 | 10 | 25 |  | Reading (covert) | 9 | 3T Bruker | SPM99 | Punc<0.001 | 3020 |  |
| (Small et al. 2009) | 12 | 3 | 9 | 62 | 55-69 | Reading (covert) on internet | 41 | 3T Allegra Siemens | FMRIB | Pcluster<0.05 | 2500 | 3.1 |
| (Ingham et al. 2012) | 12 | 12 | 0 | 35 | 20-65 | Reading | 32 | 1.9T Elscint Prestige | SPM8 | P(FDR)<0.05 |  |  |
| (Toyomura, Fujii, and Kuriki 2011) | 12 | 11 | 1 | 27 | 22-44 | Reading (overt) | 8 | 1.5T Signa GE | SPM8 | P(FWE)<0.05 | 3000 |  |
| (Aziz-Zadeh et al. 2006) | 12 | 4 | 8 | 24 | 20-37 | Reading | 7 | 3T Allegra Siemens | FMRISTAT | Punc<0.005 | 2000 | 3 |
| (Elizabeth A. Kensinger and Schacter 2005) | 16 | 8 | 8 |  | 18-30 | Reading and recall | 31 | 1.5T Sonata Siemens | SPM99 | Punc<0.001 | 3000 |  |
| (E. A. Kensinger and Corkin 2004) | 28 | 14 | 14 |  |  | Reading and encoding | 21 | 3T Allegra Siemens | SPM99 | Punc<0.001 |  |  |
| (Nieuwland, Petersson, and Van Berkum 2007) | 22 | 4 | 18 | 21 |  | Reading Sentences | 40 | 3T Philips | SPM5 | P(FDR)<0.05 | 1960 | 3 |
| (Moseley et al. 2012) | 18 |  |  | 29 |  | Reading words | 83 | 3T Trio Siemens | SPM5 | P(FDR)<0.05 | 2000 | 3 |
| (Abutalebi et al. 2007) | 12 | 0 | 12 | 25 | 22-29 | Reading | 65 | 1.5T GE | SPM2 | P(FDR)<0.01 | 2000 |  |
| (Bahlmann et al. 2007) | 12 | 3 | 9 |  | 20-28 | Reading | 5 | 1.5T Signa GE | SPM99 | Pcluster<0.001 | 1500 | 3.125 |
| (Carreiras, Mechelli, and Price 2006) | 16 | 6 | 10 |  | 22-46 | Reading words aloud | 2 | 1.5T siemens | SPM2 | Pcorr<0.05 | 3150 | 3 |
| (Clément and Belleville 2009) | 10 | 2 | 8 | 71 | 55-80 | Reading pseudowords | 1 | 3T Magnetom Siemens | SPM2 | Punc<0.001 | 2000 | 3.75 |
| (Grewe et al. 2005) | 16 | 9 | 7 | 25 | 21-32 | Reading Sentences | 7 | 3T MedSpec Bruker | LIPSIA | Punc<0.001 | 2000 |  |
| (Christodoulou et al. 2014) | 12 | 7 | 5 | 22 | 18-28 | Reading fluency task | 21 | 3T Magnetom Siemens | SPM8 | P(FDR)<0.001 | 2000 | 3.1 |
| (Toyomura, Fujii, and Kuriki 2015) | 10 | 10 | 0 | 25 | 22-33 | Reading (overt) | 18 | 1.5T Signa GE | SPM8 | P(FWE)<0.05 | 3000 | 5 |
| (Kiehl, Laurens, and Liddle 2002) | 28 | 14 | 14 | 25 |  | Reading Sentences | 12 | 1.5T Horizon GE | SPM99 |  | 3000 | 3.75 |
| (Haller et al. 2007) | 16 | 8 | 8 | 28 | 21-39 | Reading words | 14 | 1.5T Shymphony Siemens | Brainvoyager | P(FDR)<0.0001 | 3000 | 4 |
| (Rindova, Petkova, and Kotha 2007) | 12 | 8 | 4 | 23 | 19-31 | Reading Sentences | 10 | 3T MedSpec Bruker | LIPSIA | Punc<0.001 | 2000 | 3 |
| (Kinno et al. 2008) | 14 | 10 | 4 |  | 20-31 | Reading Sentences | 11 | 1.5T Stratis II Hitachi | SPM2 | P(FDR)<0.05 | 4000 | 3 |
| (Opitz and Friederici 2007) | 24 | 13 | 11 | 25 | 21-31 | Reading Words and sentences /Recall | 13 | 3T MedSpec Bruker | LIPSIA | Punc<0.0001 | 2000 | 3 |
| (Longe et al. 2007) | 12 | 3 | 9 | 25 | 20-37 | Reading / semantic discrimination | 49 | 3T MedSpec Bruker | SPM99 | Pcorr<0.05 | 1100 | 3.1 |
| (Tyler and Marslen-Wilson 2008) | 15 | 5 | 10 |  | 19-47 | Reading / semantic discrimination | 9 |  |  |  |  |  |
| (Booth et al. 2002) | 13 | 3 | 10 | 25 | 20-35 | N-back reading and semantic discrimination | 30 | 1.5T GE | SPM99 | Punc<0.001 | 3000 | 3.43 |
| (Booth et al. 2003) | 11 | 1 | 10 | 25 | 20-35 | N-back reading and semantic discrimination | 8 | 1.5T GE | SPM99 |  | 3000 | 3.43 |
| (Chee et al. 2003) | 12 | 8 | 4 |  | 19-26 | Reading / semantic discrimination | 11 | 2T Tomikon Bruker | Brainvoyager 2000 v.4.6 | Punc<0.001 | 2000 | 4 |
|  | 8 | 4 | 4 |  | 21-30 | Reading / semantic discrimination | 9 |  |  |  |  |  |
|  | 12 | 8 | 4 |  | 18-23 | Reading / semantic discrimination | 15 |  |  |  |  |  |
| (Binder et al. 2003) | 24 | 9 | 15 | 25 | 18-49 | Reading words and pseudowords | 82 | 1.5T Signa GE | AFNI | Punc<0.01 | 2000 | 3.75 |
| (Poldrack and Gabrieli 2001) | 16 | 7 | 9 | 20 |  | Reading words and pseudowords | 54 | 1.5T Signa GE | SPM99 | Punc<0.001 | 2880 | 2.9 |
| (Tagamets et al. 2000) | 11 | 6 | 5 | 28 | 20-47 | N-back reading | 107 | 1.5T Vision Siemens | SPM96 | Pcorr<0.05 | 3000 | 3.75 |
| (Kuchinke et al. 2005) | 20 | 8 | 12 | 26 | 20-36 | Reading words and pseudowords | 22 | 3T Allegra Siemens | SPM2 | Punc<0.001 | 2500 | 3 |
| (Chee et al. 1999) | 8 | 5 | 3 |  | 22-38 | Reading words | 27 | 1.5T Signa GE |  | Punc<0.00001 | 2000 | 3.125 |
| (Jessen et al. 1999) | 12 | 7 | 5 | 26,5 | 22-35 | Reading words and strings | 4 | 1.5T Vision Siemens | SPM96 | Punc<0.001 |  | 4 |
| (Robertson et al. 2000) | 8 | 4 | 4 |  |  | Reading sentences | 16 |  | SPM96 | Punc<0.05 | 3000 |  |
| (Joubert et al. 2004) | 10 | 10 | 0 | 26 |  | Reading words | 29 | 1.5T Vision Siemens | SPM96 | Punc<0.0005 |  |  |
| (Gates and Yoon 2005) | 10 | 5 | 5 | 24 | 21-32 | Reading words | 36 | 1.5T Vision Siemens | AFNI | Punc<0.001 | 5525 | 2 |
| (Mechelli et al. 2005) | 22 | 12 | 10 | 36 | 21-54 | Reading | 6 | 1.5T Siemens | SPM2 | Pcorr<0.05 | 3150 | 3 |
| (Vigneau et al. 2005) | 23 | 23 | 0 | 22,1 | 18-28 | Reading words | 17 | 1.5T Horizon GE | SPM99 | Punc<0.001 | 6000 | 3.75 |
| (Xu et al. 2005) | 22 | 22 | 0 | 34 | 21-65 | Reading sentences | 17 | 3T GE | SPM99 | Punc<0.001 | 2000 |  |
| (Valdois et al. 2006) | 20 | 14 | 6 | 23 |  | Reading | 19 | 1.5T Philips | SPM99 | voxelwise threshold p<0.001 | 2000 |  |
| (Vinckier et al. 2007) | 12 | 4 | 8 | 24 |  | Reading Words | 10 | 3T Bruker | SPM2 |  | 2400 | 3 |
| Punc: P value uncorrected; Pcorr: P value corrected for multiple comparisons; M: Male; F: Female; | | | | | | | | | | | | |

**Table A2. Calculation/arithmetic studies included in the meta-analysis.**

| **Reference** | **N** | **Gender** | | **Age (years)** | | | **Task** | | **Foci** | | **MRI vendor and Field Strength** | **Software**  **Used** | **Statistical threshold** | **TR (ms)** | **Resolution (mm)** |
| --- | --- | --- | --- | --- | --- | --- | --- | --- | --- | --- | --- | --- | --- | --- | --- |
|  |  | M | F | Mean | Range |  | |  | |  | |  |  |  |  |
| (S.M. Rivera et al. 2005) | 17 | 6 | 11 |  | 8.53-19 | Arithmetic operations | | 7 | | 1.5T Signa GE | | SPM99 | Pcluster<0.01 | 2000 | 4.35 |
| (Chassy and Grodd 2012) | 16 | 16 | 0 | 28.6 |  | Arithmetic operations | | 17 | | 3T Tim Trio Siemens | | SPM8 | Punc<0.0001 | 2000 | 4 |
| (Grabner, Ischebeck, et al. 2009) | 14 | 14 | 0 |  | 22-33 | Calculation (Multiplication) | | 63 | | 3T Tim Trio Siemens | | SPM5 | P(FWE)<0.05 | 2800 | 3 |
| (Davis et al. 2009) | 10 | 5 | 5 | 30.7 | 25-49 | Calculation | | 60 | | 3T Achieva Philips | | Brainvoyager QX | voxelwise P<0.0001 | 2000 | 3.5 |
| (Krueger et al. 2008) | 18 | 13 | 5 | 25.3 |  | Arithmetic operations | | 12 | | 1.5T GE | | SPM2 | P(FDR)<0.05 | 2000 | 3.75 |
| (Haist et al. 2015) | 16 | 8 | 8 | 22.4 | 18-34 | Calculation | | 18 | | 3T Signa GE | | AFNI | voxelwise P<0.05 | 2000 | 4 |
| (Fehr, Code, and Herrmann 2007) | 11 | 5 | 6 | 26.8 |  | Calculation | | 59 | | 1.5T Signa GE | | SPM2 | P(FDR)<0.01 | 2000 | 5 |
| (Kong et al. 2005) | 16 | 7 | 9 | 28 | 25-36 | Calculation (Addition and subtraction) | | 40 | | 3T Allegra Siemens | | SPM99 | Punc<0.001 | 2500 | 3.13 |
| (Wu et al. 2009) | 18 | 7 | 11 | 22.3 | 18-31.5 | Calculation vs identification | | 7 | | 3T Signa GE | | SPM5 | voxelwise  threshold P < 0.01) | 2000 | 3.125 |
| (Ischebeck et al. 2009) | 17 | 10 | 7 | 25 |  | Calculation (Multiplication) | | 19 | | 1.5T Symphony Siemens | | SPM2 | Pcluster<0.05 | 3000 | 3 |
| (Kaufmann et al. 2008) | 12 | 6 | 6 | 33.2 |  | Arithmetic operations | | 5 | | 1.5T Magnetom Siemens | | SPM2 | Punc<0.001 | 3060 | 3.2 |
| (Notebaert, Pesenti, and Reynvoet 2009) | 13 | 13 | 0 |  | 19-30 | Number identification | | 22 | | 3T Intera Philips | | SPM5 | Pcorr<0.001 | 2000 | 2.75 |
| (Philippe Pinel et al. 2001) | 13 |  |  | 25 |  | Number comparison | | 16 | | 3T Bruker | | SPM99 | Pcorr<0.05 | 2000 | 3 |
| (Holloway, Price, and Ansari 2010) | 19 | 9 | 10 | 24 | 18-28 | Arithmetic operations | | 15 | | 3T Intera Philips | | Brainvoyager QX 2.0.7 | Punc<0.005 | 3000 |  |
| (Le Clec’H et al. 2000) | 11 |  |  |  | 23-37 | Number comparison | | 7 | | 3T Bruker | | SPM96 | voxelwise P<0.001 | 2000 | 5 |
| (Reinecke et al. 2012) | 10 | 3 | 7 | 25 | 22-34 | Calculation (subtraction) | | 24 | | 1.5T Signa Siemens | | SPM99 | voxelwise P<0.01 | 2000 | 3.75 |
| (Chochon et al. 1999) | 8 | 4 | 4 | 25 | 20-30 | Calculation and number comparison | | 56 | | 3T Bruker | | SPM96 | Pcorr<0.05 | 6000 | 5 |
| (S Dehaene et al. 1999) | 8 | 4 | 4 | 25 | 22-28 | Calculation (addition) | | 36 | | 3T Bruker | | SPM96 | Pcorr<0.05 | 4000 | 5 |
| (Delazer et al. 2003) | 13 | 7 | 6 | 30.5 |  | Calculation (Multiplication) | | 21 | | 1.5T Vision Siemens | | SPM99b | voxelwise P<0.001 | 3,75 |  |
| (K. M. Lee 2000) | 11 | 6 | 5 |  | 25-35 | Calculation (Multiplication and subtraction) | | 14 | |  | |  |  |  |  |
| (Naccache and Dehaene 2001) | 9 | 7 | 2 | 26 |  | Number comparison | | 2 | | 3T Bruker | | SPM96 | Pcorr<0.05 | 2000 | 3 |
| (Hugdahl et al. 2004) | 12 | 5 | 7 | 31 |  | Calculation | | 4 | | 1.5T Vision Plus Siemens | | SPM99 |  | 6000 | 3.44 |
| (Garavan et al. 2000) | 11 | 7 | 4 | 29 | 19-41 | Counting | | 40 | | 1.5T Signa GE | | AFNIv2.2 | Punc<0.01 | 4800 | 3.75 |
| (Simon et al. 2004) | 10 | 3 | 7 | 25 | 22-34 | Calculation | | 11 | | 1.5T Signa GE | | SPM99 | Punc<0.01 | 2000 | 3.75 |
| (Lazeron et al. 2003) | 9 | 6 | 3 | 24 | 19-30 | Calculation (addition) | | 21 | | 1.5T | | SPM99b | Pcorr<0.05 | 4000 | 3.44 |
| (H.-J. Lee et al. 2007) | 15 | 7 | 8 | 32 |  | Counting | | 7 | | 1.5T Sonata Siemens | | SPM2 | P(FWE)<0.05 | 2600 | 3.4 |
| (Ischebeck et al. 2006) | 12 | 4 | 8 | 27 |  | Calculation (Multiplication and subtraction) | | 60 | | 1.5T Sonata Siemens | | SPM2 | Pcluster<0.05 | 3000 | 4 |
| (Blackwood et al. 2004) | 8 | 8 | 0 | 38 | 18-53 | Counting | | 10 | | 1.5T Signa GE | | SPM99 | Pcorr<0.05 | 3000 | 3.75 |
| (Susan M. Rivera et al. 2002) | 16 | 0 | 16 | 17 | 11-23 | Arithmetic operations | | 21 | | 1.5T Signa GE | | SPM99 | Pcorr<0.05 | 2000 | 3.75 |
| (Newman, Willoughby, and Pruce 2011) | 15 | 6 | 9 | 22 | 18-31 | Arithmetic operations | | 41 | | 3T Tim Trio Siemens | | SPM5 | P(FWE)<0.05 | 1000 | 3.125 |
| (Grabner et al. 2007) | 12 |  |  | 26 | 22-32 | Arithmetic operations | | 16 | | 3T Tim Trio Siemens | | SPM5 | Punc<0.0001 | 2800 | 3 |
| (Grabner, Ansari, et al. 2009) | 28 | 28 | 0 | 27 | 22-33 | Arithmetic operations | | 10 | | 3T Tim Trio Siemens | | SPM5 | Punc<0.001 | 2000 | 3 |
| (Maruishi et al. 2007) | 12 |  |  | 26 |  | Calculation (Addition) | | 7 | | 1.5T Symphony Siemens | | SPM2 | Pcorr<0.05 | 2200 | 3 |
| (Hayashi et al. 2013) | 26 | 12 | 14 |  | 19-30 | Counting/Calculation | | 28 | | 3T Allegra Siemens | | SPM8 |  | 2000 | 3.5 |
| (He et al. 2014) | 20 | 12 | 8 | 21 |  | Number comparison | | 22 | | 7T Siemens | | SPM8 | P(FWE)<0.05 | 2000 | 1.4 |
| (Holloway and Ansari 2010) | 19 | 9 | 10 | 23 | 18-28 | Number comparison | | 5 | | 3T Intera Philips | | BrainVoyager QX 1.10.4 | Pbonferroni<0.01 | 3000 | 4 |
| (Kaufmann et al. 2005) | 17 | 10 | 7 | 31 | 25-42 | Number comparison | | 25 | | 1.5T Vision Siemens | | SPM99 | Punc<0.005 | 2000 | 3.91 |
| (X. Liu et al. 2006) | 12 | 5 | 7 |  | 18-45 | Number comparison | | 35 | | 3T Tim Trio Siemens | | FMRIB’s |  | 2000 | 3.5 |
| (Piazza et al. 2007) | 14 |  |  |  |  | Calculation | | 6 | | 3T Bruker | | SPM2 | Pcluster<0.05 | 2400 | 3 |
| (Venkatraman, Ansari, and Chee 2005) | 10 |  |  |  | 20-25 | Counting/Calculation | | 51 | | 3T Allegra Siemens | | Brainvoyager2000 | Punc<0.001 | 3000 | 3 |
| (Bulthé, De Smedt, and Op de Beeck 2014) | 14 | 2 | 12 |  | 21-28 | Calculation (subtraction) | | 19 | | 3T Intera Philips | | SPM8 |  | 3000 | 2.1 |
| (Eger et al. 2009) | 10 | 5 | 5 | 23 |  | Counting/Calculation | | 18 | | 3T Tim Trio Siemens | | SPM5 |  | 2500 | 1.5 |
| (Göbel et al. 2004) | 12 | 6 | 6 | 27 | 20-34 | Number comparison | | 37 | | 3T Inova Varian | | FMRIB FSL | Pcluster<0.01 | 3000 | 5 |
| (P Pinel et al. 1999) | 11 | 9 | 2 | 26 |  | Number comparison | | 48 | | 3T Bruker | | SPM96 | Pcorr<0.05 | 2000 | 4 |
| (Santens et al. 2010) | 16 | 14 | 2 | 22 | 19-26 | Counting/Calculation | | 6 | | 3T Magnetom Trio Siemens | | SPM5 | Punc<0.005 | 2000 | 3 |
| (Santens et al. 2010) | 12 | 12 | 0 | 20 | 19-23 | Counting/Calculation | | 2 | | 3T Magnetom Trio Siemens | | SPM5 | Punc<0.005 | 2000 | 3 |
| (Gandini et al. 2008) | 13 | 7 | 6 | 26 | 23-30 | Arithmetic operations | | 11 | | 3T Bruker | | SPM5 | Punc<0.001 | 2916 | 3 |
| (Kucian et al. 2006) | 20 | 10 | 10 | 26 |  | Calculation and magnitude comparison | | 42 | | 1.5T GE | | SPM99 | P(FDR)<0.05 | 3200 | 3.75 |
| (Wood, Nuerk, and Willmes 2006) | 14 | 14 | 0 | 27 | 21-38 | Magnitude Comparison | | 22 | | 1.5T Gyroscan Philips | | SPM2 | Punc<0.001 | 2800 | 3.4 |
| (Tully, Lincoln, and Hooker 2014) | 24 |  |  | 35 | 19-55 | Counting/Calculation | | 22 | | 3T Tim Trio Siemens | | SPM8 | Punc<0.001 |  |  |
| (Armbruster et al. 2012) | 20 | 10 | 10 | 23 | 20-32 | Number comparison | | 37 | | 3T Trio Siemens | | SPM8 | Punc<0.005 | 2000 | 3 |
| (Ischebeck et al. 2008) | 19 | 8 | 11 | 28 |  | Arithmetic operations | | 11 | | 1.5T Symphony Siemens | | SPM2 | P(FWE)<0.001 | 3000 | 3 |
| (Philippe Pinel et al. 2004) | 15 | 6 | 9 | 24 |  | Arithmetic operations | | 28 | | 3T Bruker | | SPM99 | Pcorr<0.05 | 2400 | 4.5 |
| (Cantlon et al. 2009) | 14 | 8 | 6 | 24 |  | Magnitude comparison | | 6 | | 3T Signa Excite GE | | SPM2 | Pcluster<0.05 | 2000 | 4 |
| (Piazza et al. 2006) | 10 | 7 | 3 |  | 23-31 | Counting and Magnitude comparison | | 30 | | 2T Vision Siemens | | SPM99 | Punc<0.05 | 2974 | 2.5 |
| (Ansari et al. 2005) | 12 |  |  | 19 | 19-21 | Number comparison | | 12 | | 1.5T GE | | Brainvoyager QX 1.2.8 | Punc<0.005 | 2500 |  |
| (Ansari, Dhital, and Siong 2006) | 16 | 16 | 0 | 20 |  | Magnitude comparison | | 8 | | 1.5T GE | | Brainvoyager QX 1.2.6 | Punc<0.0001 | 2500 | 4.5 |
| (Ansari and Dhital 2006) | 9 | 6 | 3 | 19 | 19-21 | Magnitude comparison | | 7 | | 1.5T GE | | Brainvoyager QX 1.2.8 | Punc<0.001 | 2500 | 4.5 |
| (Ansari et al. 2007) | 13 |  |  | 21 | 19-27 | Estimation and counting, Magnitude comparison | | 14 | | 3T Intera Philips | | Brainvoyager QX 1.7 | P(FDR)<0.05 | 2500 | 4 |
| (Vogel et al. 2013) | 14 | 7 | 7 | 25 | 18-33 | Arithmetic operations | | 15 | | 3T GE | | Brainvoyager QX 2.3 | Punc<0.001 | 2100 | 3.4 |
| (Leibovich et al. 2016) | 19 | 7 | 12 | 23 |  | Magnitude comparison | | 12 | | 3T Tim Trio Siemens | | Brainvoyager QX 2.4 | Punc<0.005 | 2000 | 3 |
| (Chen et al. 2007) | 20 | 10 | 10 | 23 | 18-30 | Arithmetic operations | | 8 | | 3T Elscint Prestige GE | | SPM2 | Punc<0.001 | 2000 | 3.75 |
| (V Menon et al. 2000) | 16 | 8 | 8 | 20.3 | 17-23 | Calculation (3 operands) | | 27 | | 1.5T Signa GE | | SPM99 | Pcorr<0.01 | 2000 | 4.35 |
| (Rickard et al. 2000) | 8 | 3 | 5 | 24 | 20-34 | Calculation (multiplication) | | 8 | | 1.5T Signa GE | | SPM96b | Punc<0.001 | 3000 |  |
| (Vinod Menon et al. 2002) | 16 | 8 | 8 | 19.5 | 16-23 | Arithmetic operations | | 2 | | 1.5T Signa GE | | SPM99 | Pcorr<0.01 | 2000 | 3.75 |
| (Kawashima et al. 2004) | 8 | 4 | 4 | 44.1 | 40-49 | Arithmetic operations | | 28 | | 1.5T Symphony Siemens | | SPM99b | Pcorr<0.05 | 4000 | 3 |
| (Molko et al. 2003) | 14 |  |  | 24.3 |  | Calculation | | 11 | | 1.5T Signa GE | | SPM99 | voxelwise P<0.001 | 4000 | 3.75 |
| (De Pisapia, Slomski, and Braver 2006) | 20 | 8 | 12 | 21,5 |  | Calculation | | 9 | | 1.5T Vision Siemens | |  | Punc<0.05 | 2500 | 3.75 |
| (Zhou et al. 2007) | 20 | 10 | 10 | 22.7 | 18-30 | Calculation (Multiplication) | | 14 | | 3T Excite GE | | SPM2 | Punc<0.001 | 3000 | 3.75 |
| (Kuo et al. 2008) | 12 | 6 | 6 | 25 | 21-29 | Calculation (Addition) | | 21 | | 3T MedSpec Bruker | | SPM99 | voxelwise P<0.001 | 3000 |  |
| (Jost et al. 2009) | 16 | 6 | 10 | 24,5 | 20-30 | Calculation (Multiplication) | | 5 | | 1.5T Signa GE | | SPM2 | Punc<0.001 | 2000 | 3.75 |
| (Keller and Menon 2009) | 49 | 24 | 25 | 23.99 | 18-36 | Calculation | | 9 | | 3T Signa GE | | SPM5 | Pclust<0.01 | 2000 | 3.125 |
| (Andres et al. 2011) | 10 | 10 | 0 | 21 | 19-23 | Calculation (Multiplication and Subtraction) | | 8 | | 3T Achieva Philips | | SPM2 | P(FWE)<0.05 | 2500 | 3.5 |
| Punc: P value uncorrected; Pcorr: P value corrected for multiple comparisons; M: Male; F: Female; | | | | | | | | | | | | | | | |

**Table A3. Major Activation likelihood estimation results for the reading, calculation and programming tasks separate analysis.**

| **Task** | **Cluster** | **Peak coords** | | | **ALE** | **Hem.** | **Lobe** | **Region** | **BA** |
| --- | --- | --- | --- | --- | --- | --- | --- | --- | --- |
|  |  | **X** | **Y** | **Z** |  |  |  |  |  |
| **Reading** | 1 | -47.99 | 23.74 | 16.8 | 0.09909219 | L | Frontal | Middle Frontal Gyrus | 46 |
| P(FWE)<0.05 (1000 permutations) |  | -45.68 | 5.74 | 31.66 | 0.08507671 | L | Frontal | Inferior Frontal Gyrus | 6 |
| Cluster Threshold>200 |  | -47.68 | 0.58 | 47.73 | 0.06985907 | L | Frontal | Precentral Gyrus | 6 |
|  |  | -43.87 | 26.62 | -1.34 | 0.052802455 | L | Frontal | Inferior Frontal Gyrus | 13 |
|  |  | -54.12 | -10.71 | 39.83 | 0.045808453 | L | Frontal | Postcentral Gyrus | 4 |
|  |  | -52.02 | -8.93 | 35.2 | 0.045547023 | L | Frontal | Precentral Gyrus | 6 |
|  | 2 | -43.7 | -59.51 | -14.19 | 0.07579479 | L | Temporal | Fusiform Gyrus | 37 |
|  |  | -41.62 | -49.1 | -17.32 | 0.07101602 | L | Temporal | Fusiform Gyrus | 37 |
|  | 3 | -.62 | 7.9 | 55.5 | 0.0694997 | L | Frontal | Medial Frontal Gyrus | 6 |
|  |  | 5.69 | 15.71 | 45.85 | 0.060160756 | R | Frontal | Medial Frontal Gyrus | 32 |
|  | 4 | 37.32 | 26.76 | -6.74 | 0.053327434 | R | Frontal | Inferior Frontal Gyrus | 47 |
|  | 5 | 50.37 | 24.84 | 22.27 | 0.053470768 | R | Frontal | Middle Frontal Gyrus | 46 |
|  |  |  |  |  |  |  |  |  |  |
| **Calculation** | 1 | 31.54 | -52.33 | 44.57 | 0.10145992 | R | Parietal | Superior Parietal Lobule | 7 |
| P(FWE)<0.05 (1000 permutations) |  | 48.6 | -32.85 | 47.2 | 0.06980936 | R | Parietal | Inferior Parietal Lobule | 40 |
| Cluster Threshold>200 | 2 | -30.43 | -50.41 | 47.35 | 0.07494718 | L | Parietal | Superior Parietal Lobule | 7 |
|  |  | -28.26 | -58.86 | 48.03 | 0.06795954 | L | Parietal | Superior Parietal Lobule | 7 |
|  |  | -45.45 | -37.98 | 44.25 | 0.0644993 | L | Parietal | Inferior Parietal Lobule | 40 |
|  | 3 | -4.98 | 15.82 | 48.19 | 0.07429182 | L | Frontal | Superior Frontal Gyrus | 6 |
|  |  | 5.73 | 13.94 | 50.46 | 0.072859675 | R | Frontal | Superior Frontal Gyrus | 6 |
|  | 4 | -45.71 | 7.69 | 29.27 | 0.08323778 | L | Frontal | Inferior Frontal Gyrus | 9 |
|  | 5 | 48.29 | 14.42 | 25.39 | 0.07794736 | R | Frontal | Inferior Frontal Gyrus | 9 |
|  | 6 | 35.23 | 22.86 | -1.93 | 0.07916224 | R | Sub-lobar | Insula | 13 |
|  | 7 | -33.15 | 22.63 | 1.11 | 0.05432502 | L | Sub-lobar | Insula | 13 |
|  | 8 | -26.24 | -3 | 54.48 | 0.0652489 | L | Frontal | Middle Frontal Gyrus | 6 |
|  | 9 | -43.68 | -68.15 | -15.69 | 0.04688309 | L |  | Cerebellum Posterior Lobe | * |
|  | 10 | -45.83 | 30.61 | 22.89 | 0.04818503 | L | Frontal | Middle Frontal Gyrus | 9 |
|  | 11 | 46.02 | 39.44 | 18.86 | 0.044800203 | R | Frontal | Middle Frontal Gyrus | 46 |
|  |  |  |  |  |  |  |  |  |  |
| **Programming** | 1 | 46.46 | 22.24 | 9.88 | 0.008191813 | R | Frontal | Inferior Frontal Gyrus | 45 |
| P<0.01 (1000 permutations) |  | 38.38 | 16.27 | 5.212 | 0.007993459 | R | Sub-lobar | Insula | 13 |
| Cluster Threshold>200 | 2 | -50.51 | 27.49 | 29.75 | 0.010981998 | L | Frontal | Middle Frontal Gyrus | 46 |
|  | 3 | -50.51 | -55.6 | -3.214 | 0.008036627 | L | Temporal | Middle Temporal Gyrus | 37 |
|  | 4 | -30.3 | -73.51 | 26.59 | 0.008036611 | L | Occipital | Superior Occipital Gyrus | 19 |
|  | 5 | 24.24 | 1.026 | 65.35 | 0.008036611 | R | Frontal | Middle Frontal Gyrus | 6 |
|  | 6 | -57.58 | -15.91 | -14.03 | 0.007545747 | L | Temporal | Middle Temporal Gyrus | 21 |
|  | 7 | -12.12 | -3.402 | 71.65 | 0.008036611 | L | Frontal | Superior Frontal Gyrus | 6 |
|  | 8 | -60.61 | -40.67 | -14.27 | 0.007787343 | L | Temporal | Middle Temporal Gyrus | 21 |
|  | 9 | 30.3 | -81.85 | 6.562 | 0.007787312 | R | Occipital | Middle Occipital Gyrus | 19 |
|  | 10 | -42.42 | -2.782 | 36.85 | 0.007787312 | L | Frontal | Precentral Gyrus | 6 |
|  | 11 | 48.48 | 3.086 | 43.69 | 0.007787312 | R | Frontal | Middle Frontal Gyrus | 6 |
|  | 12 | -52.53 | -74.23 | -1.907 | 0.008036611 | L | Occipital | Inferior Temporal Gyrus | 37 |
|  | 13 | 6.061 | 51.37 | 48.42 | 0.008036611 | R | Frontal | Medial Frontal Gyrus | 8 |
|  | 14 | -54.55 | -31.41 | 52.76 | 0.007084864 | L | Parietal | Postcentral Gyrus | 40 |
|  | 15 | 50.51 | 27.08 | 38.43 | 0.007545747 | R | Frontal | Middle Frontal Gyrus | 9 |
